# Multidimensional Sternal Fixation to Overcome a “Floating” Sternum

**DOI:** 10.1155/2014/690160

**Published:** 2014-10-14

**Authors:** William Rothstein, Tyler Spata, Bryan Whitson, Ahmet Kilic

**Affiliations:** ^1^Department of Surgery, The Ohio State University Wexner Medical Center, 410 W. 10th Avenue, Columbus, OH 43210, USA; ^2^Division of Cardiac Surgery, Department of Surgery, The Ohio State University Wexner Medical Center, 410 W. 10th Avenue, Columbus, OH 43210, USA

## Abstract

This case report describes the repair of a complete sternal dehiscence of the lower right sternum using sternal wires, manubrial plates, and a Talon closure device for rigid, multidimensional sternal fixation. Sternal dehiscence is a rare but significant cause of morbidity for patients undergoing median sternotomy. The risk factors for this complication are well described and although sternal wires have traditionally been used for primary closure, rigid fixation with sternal plates is a viable alternative to avoid dehiscence in this high-risk cohort.

## 1. Introduction

The median sternotomy is the most commonly used incision for open cardiac surgery due to the ease of access to the heart and surrounding structures [1]. Postoperative sternal dehiscence occurs in 0.4% of patients, requiring a reoperation on average 5.4 months postoperatively [2]. In addition to causing significant pain for the patient, it also predisposes patients to develop eventual mediastinitis, resulting in a high morbidity for the patient [3]. Therefore, effective primary closure of sternal dehiscence is important in preventing high-risk complications and patient discomfort. Traditionally, sternal wires have been used to primarily close sternal dehiscence; however, sternal plates are becoming increasingly used as an alternative. In this report, we describe the use of a combination of sternal rewiring, a manubrial plate, and a Talon closure device for rigid, multidimensional sternal fixation in a patient with an unstable “floating” sternum.

## 2. Case Report

A seventy-two-year-old male was referred from an outside hospital for an unstable sternum five months after undergoing a two-vessel coronary artery bypass graft. During the patient's postoperative course, he suffered from significant anxiety and panic attacks, causing him to move uncontrollably. Four days after his original operation, he underwent a sternal rewiring procedure for an unstable sternum. After his discharge, the patient reported having discomfort in his sternum with coughing resulting in recurrent vigorous coughs and feelings of doom and shortness of breath. He complained of feeling his wires move and the lower part of his sternum moving with each breath. Additionally, he stated that the pain from his sternum limited his mobility and was mentally debilitating preventing him from enjoying any quality of life.

**Figure 1:**
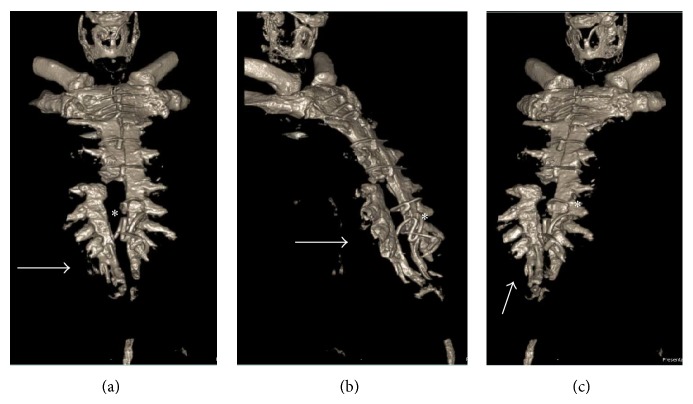
Preoperative 3-dimensional computed tomography showing the right lower part of the “floating” sternum. (a) Anterior-posterior view, (b) right anterior-oblique view, and (c) left anterior-oblique view showing the dehisced right lower part of sternum (highlighted with an* arrow*) with sternal wires that have pulled through (highlighted with an* asterisk*).

The patient's past medical history was significant for his coronary arterial disease, congestive heart failure, mitral regurgitation, gout, and obesity (body mass index of 35.6 kg/m^2^). On physical exam, inspection of his chest wall revealed paradoxical movement of the lower right part of his sternum that was more easily produced with Valsalva maneuvers. Preoperative computed tomography (CT) with 3D reconstruction (Figure 1) confirmed complete sternal dehiscence of the lower right sternum. The uppermost hemisternum had healed well with four stable, intact interrupted sternal wires.

**Figure 2:**
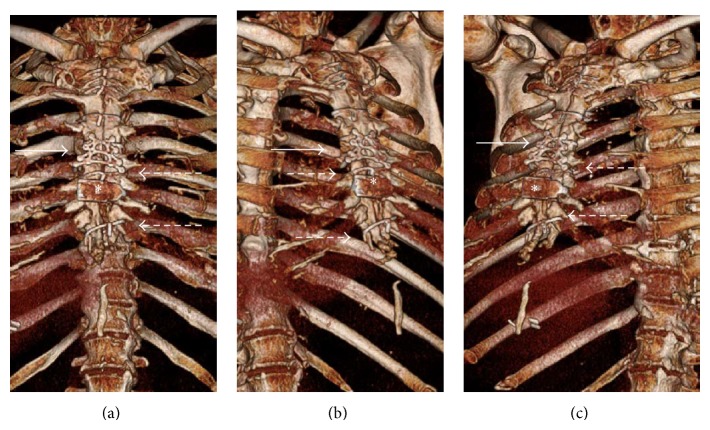
One-month postoperative 3-dimensional computed tomography showing rigid fixation of the “floating” sternum. (a) Anterior-posterior view, (b) right anterior-oblique view, and (c) left anterior-oblique view showing the completed reconstruction. The sternum is intact with appropriate healing. The A-shaped manubrial plate is highlighted by* solid arrow*, the sternal closure device is highlighted by an* asterisk,* and the simple sternal wires are highlighted by* dashed arrows*.

After informed consent, the patient underwent a partial redo sternotomy with sharp debridement of the lower part of his sternum. Given the “floating” nature of the caudal right part of his sternum, we elected to perform a multidimensional fixation to ensure optimal outcome. To prevent any horizontal movement, we placed interrupted sternal wires along with a Talon closure device (KLS Martin, Jacksonville, FL). To further prevent any craniocaudal dehiscence and promoting healing of the fractured right hemisternum, we screwed in a pyramidal “A”-shaped manubrial plate (KLS Martin, Jacksonville, FL). The patient had an uncomplicated postoperative course and was discharged home on post-op day number two with sternal precautions. He returned to clinic in 3 weeks without any complaints. Physical examination and confirmatory chest CT with 3D reconstruction (Figure 2) showed an intact sternum with appropriate healing.

## 3. Discussion

Sternal complications following a median sternotomy can occur between 0.5 and 5% of all patients acting as a source of significant morbidity for these patients [2, 3]. Known preoperative risk factors for frank sternal dehiscence include heart failure, obesity, chronic obstructive pulmonary disease, frequent coughing, malnutrition, age, tobacco use, diabetes, and immunosuppression [3–5]. Perioperative risk factors include prolonged operative time, excessive use of electrocautery, bilateral mammary artery usage, utilization of cardiopulmonary bypass, postoperative bleeding and need for blood product transfusions, chest compressions, and reoperation [5, 6]. Dehiscence can occur if the edges of the sternum are not aligned properly, the sternum is ischemic, or the bone is abnormal and/or osteopenic [2, 7, 8].

Sternal rewiring is the most common way of primary closure for sternal dehiscence; however, sternal closure devices and/or plating in patients with multiple risk factors are being used more commonly. The Sternal Talon closure device is made of biocompatible titanium as to allow sternal closure without bone penetration. The theoretical advantage of the design is to allow for a more even distribution of horizontal forces along the sternal surface [9–11]. Indeed, even a single plate along with sternal wire closure improves the strength potentially reducing the risk of sternal dehiscence in cadaveric studies [12]. However, caution should be made in the off-midline sternotomy and in cases with osteopenia [13, 14]. Although one study showed enhanced postoperative recovery with the Talon device, there was no appreciable change in postoperative incentive spirometry values [15].

In other studies, rigid plate fixation as a primary closure has been shown to significantly decrease the incidence of postoperative mediastinitis compared to sternal wire closure alone [16]. Although small studies have shown a decreased length of stay without any change in the risk of sternal wound complications with primary use of sternal plates and fixation devices, we would only advocate their use in selected, high-risk patients with multiple comorbidities [5, 17, 18].

In the present case we had numerous considerations to ponder. The patient had a number of risk factors for repeat dehiscence along with the fact that he had failed an attempt at sternal rewiring. There was significant craniocaudal displacement of the sternal fracture combined with the lateral dehiscence of the sternum. Citing cadaveric studies where lateral and posteriorly placed reinforcement significantly improved sternal stability compared to six interrupted sternal wires, we elected to use a multidimensional approach to ensure optimal sternal healing [19]. Indeed, this case highlights the technology offered by sternal plates and closure devices as promising alternatives to more traditional sternal wires for repair of this complication. Additionally, consideration can be made for primary closure of the patient with multiple well-described preoperative risk factors [2–5]. Vigilant preoperative risk assessment and selective rigid sternal fixation of high-risk patients using steel plates can potentially avoid these unpleasant complications before they occur.
